# Detection and quantification of bacterial species DNA in bovine digital dermatitis lesions in swabs and fine-needle aspiration versus biopsies

**DOI:** 10.3389/fvets.2022.1040988

**Published:** 2022-11-18

**Authors:** Angelica Petersen Dias, Jeroen De Buck

**Affiliations:** Faculty of Veterinary Medicine, University of Calgary, Calgary, AB, Canada

**Keywords:** hairy heel warts, cattle, qPCR (quantitative PCR), *Treponema*, test agreement, sampling techniques, dairy cows

## Abstract

Digital Dermatitis (DD) is a polymicrobial disease characterized by ulcerative lesions on the heel bulb of cattle and for which, despite being reported almost 50 years ago, information on the causative agent is still lacking. Tissue biopsies are regularly collected to identify bacterial presence-absence and their relative abundance in the microbiome, with sufficient evidence for the high abundance of species of *Treponema* spp. and other anaerobes in lesions. However, it is unclear what the potential of less-invasive sampling methods is for bacterial detection and quantification. This study aimed to test whether less-invasive sampling techniques, such as swabs and fine-needle aspiration (FNA), can be a convenient alternative to tissue biopsies in detecting and quantifying seven DD-associated bacteria in active, ulcerative DD lesions by qPCR. Twenty-two M2 DD lesions were collected using corresponding swabs, aspirates, and biopsies from dairy cows. Presence/absence and quantities of *Treponema phagedenis, Treponema medium, Treponema pedis, Porphryromonas levii, Bacteroides pyogenes, Fusobacterium necrophorum*, and *Fusobacterium mortiferum* were correlated, and Bland-Altman plot, McNemar's test, and Cohen's kappa coefficient were used to calculate the agreement among the methods. The quantities of all species were larger in swabs and smaller in aspirates compared to biopsies; however, the differences in bacterial enumeration observed between biopsies and swabs were smaller than in biopsies and aspirates. A strong correlation was observed between the quantity of *T. pedis, T. medium, P. levii*, and *F. mortiferum* in biopsies, swabs, and FNA. Yet, *T. phagedenis* presented the smallest difference between biopsies and swabs, followed by *T. pedis* and *T. medium*. In conclusion, swabs, aspirates, and biopsies were equal in their capacity to detect *Treponema* species based on the good agreement for bacteria presence/absence, with a more limited agreement for the other anaerobes, which were more often present in M2 lesions swabs by qPCR. Bacterial numbers were higher in swabs and lower in aspirates compared to biopsies, with the amounts of treponemes in swabs being closer to biopsies than in aspirates to biopsies. Therefore, aspirates were less suitable for bacterial quantification in DD lesions compared to the other methods.

## Introduction

Digital Dermatitis (DD) is a polymicrobial infectious disease that causes skin erosion on the heel bulbs of cattle, but its precise etiology is still unclear. It is one of the main causes of lameness in dairy cattle, compromising animal welfare and leading to direct and indirect economic losses (1–3). DD can be presented either as an acute and ulcerative lesion or as chronic and proliferative. Due to the fastidious nature of the organisms involved in DD, culture-independent molecular approaches have been used to identify certain DD-associated bacteria as important constituents of the DD microbiome that are not as abundant in healthy skin. *Treponema* spp. are suggested to play a crucial role in this disease because of their dominance in the bacterial population in DD lesions, with the most common species being *T. phagedenis, T. medium*, and *T. pedis* (4). Other anaerobes are frequently identified in lesions alongside *Treponema* spp., such as *Porphyromonas, Mycoplasma, Fusobacterium, and Bacteroides* spp. (5–7). However, the exact contribution of those bacterial species to lesion development and progression is not fully understood.

DD microbiome studies mostly report bacterial relative abundance and community structure and changes; however, few investigations have used quantitative methods and applied this knowledge to assess the role of different microorganisms in disease initiation and progression at the species level. Recently, real-time polymerase chain reactions (qPCR) assays have been developed to detect and quantify DD-associated bacteria (4, 5). Therefore, the density of microorganisms, patterns of prevalence, and variation can be determined and related to lesion initiation, development, progression, and chronicity, targeting specific species. For this, tissue biopsies have been commonly used. As an invasive sampling method, biopsies require local anesthesia and can lead to bleeding, scarring, and secondary infections (8), making it difficult to sample multiple sites on the same animal or the same site in long-term repeated-sampling studies. Although some *Treponema* phylotypes were detected more deeply in DD lesions based on fluorescent *in situ* hybridization (FISH) (9) and Steiner silver staining (10), they were also detected in stratum spinosum by Steiner silver stain (11), on hoof trimmers gloves for up to 3 days in the air by PCR and culture (12), and in superficial biopsies (top 2 mm) by 16S rRNA gene sequencing (13), demonstrating also more superficial presence. Thus, less-invasive sampling techniques, such as swabs and fine-needle aspiration (FNA), might be an alternative to sampling DD lesions helping to answer remaining questions about DD microbiota.

There is sufficient evidence for the presence and high abundance of species of *Treponema* spp. and other anaerobes in DD lesions*;* however, hitherto, is unclear what is the potential of less-invasive sampling methods for bacterial detection and quantification. Therefore, this study aimed to test whether swabs and FNA can be a convenient and reliable alternative to tissue biopsies in detecting and quantifying seven DD-associated bacteria at the species level by qPCR.

## Materials and methods

### Sampling collection and processing

Holstein dairy cattle from three commercial dairy farms in central and southern Alberta, Canada, with active ulcerative DD lesions that were more than 2 cm in diameter (described as stage M2) (11) were sampled. Milking cows were screened for M2 lesions in the milking parlor using a small mirror glued to a plastic kitchen spatula for foot inspection (14, 15) and their lesions were confirmed in the trimming chute. Corresponding biopsies, aspirates, and swabs from a total of 22 M2 lesions were collected. All animal use was approved by the University of Calgary Veterinary Services Animal Care Committee (VSACC) under animal care protocol #AC21-0146. Written informed consent was obtained from the owners for the participation of their animals in this study.

A swab followed by a FNA and a biopsy were collected from the same M2 lesion at the same time point. Once the cow was restrained in the trimming chute, the lesion was cleaned with tap water and dried off with paper towels to confirm the M2 stage based on lesion appearance and size. Local anesthesia was performed before sampling with 3 mL of lidocaine subcutaneously (Lidocaine HCl 2%, Zoetis Canada Inc., Kirkland, QC). The lesion was checked to be dried before sampling to avoid water absorption during the procedure, ensuring that the absorbed material was from the lesion itself. First, the tip of a dry sterile cotton swab was positioned in the center of the lesion at 90 degrees from the lesion surface and rotated for 10 sec with sufficient pressure to absorb fluid from the lesion and immediately placed into an anaerobic transport medium (ATM, Starswab Anaerobic Transport System, Etobicoke, ON). Then, FNA was performed by carefully introducing a 22G needle attached to an empty 10 mL syringe into the lesion to a depth of around 5 mm. Once the needle was inserted, 2 to 5 mL of negative pressure was applied by briskly withdrawing the plunger multiple times, until fluid appeared at the needle hub. The aspirate was immediately injected into ATM. Finally, one biopsy was obtained using a disposable 4 mm biopsy punch (Integra LifeSciences, Princeton, NJ) from the outer margin of the lesion, as regularly performed in previous studies (4, 6). Extra biopsies (*n* = 11) were collected from the center of the lesions, in addition to the edge, from the same lesion, to test which would be more coherent with swabs. All samples were transported in ATM, which supports bacteria viability, preventing replication during transportation at ambient temperature.

In the lab, specimens were removed from the anaerobic media tubes and were processed under anaerobic conditions (5% CO_2_, 5% H_2_, 90% N_2_) in an anaerobic chamber (Bactron3000, Sheldon Manufacturing, Inc.; Cornelius, OR). Tissue biopsies were sectioned longitudinally, without removing the outer layer of the epidermal skin, and up to 25 mg were processed. Bacterial DNA was extracted using the Qiagen DNeasy Blood and Tissue kit (Qiagen, Hilden, Germany) with minor modifications. Up to 25 mg of biopsy, up to 25 mg of aspirates, recovered from ATM using a sterile disposable inoculating loop, and swab cotton heads were placed in a 1.5 mL microcentrifuge tube with 40 μL of proteinase K and 180 μL of tissue lysis (ATL) buffer and incubated at 56°C overnight. Then, the manufacturer's recommendation was followed, with the swabs being processed in the same manner as the biopsies and aspirates except for their removal prior to the ethanol addition step, with the DNA being eluted in 100 μL of DNase/RNase-free water. Extraction controls were performed by following every step of the Qiagen kit without samples. DNA was stored at−20°C until their use in qPCR.

### Quantitative polymerase chain reaction (qPCR)

Quantitative real-time PCR (CFX96 real-time system; Rio-Rad Laboratories Inc., Hercules, CA) was carried out for absolute quantification of species-specific gene copy numbers targeting seven clinically relevant DD-associated bacteria. Three different qPCR assays were conducted in this study: a multiplex qPCR developed by Beninger, et al. (4) targeting *T. phagedenis, T. medium*, and *T. pedis*, a multiplex qPCR developed by Caddey, et al. (5) for non-*Treponema* species targeting *P. levii, B. pyogenes*, and *Fusobacterium* sp. (97% identity with *Fusobacterium mortiferum*) (c), and a singleplex qPCR developed by Witcomb, et al. (16) targeting *F. necrophorum*. Those bacterial species were chosen because their identification as commonly detected organisms in bovine DD lesions in Alberta (4, 5). Primers and fluorescent probes for each reaction are shown in Table 1. For DNA quantification, sample threshold cycle (Ct) values were compared to a standard curve produced from 10-fold serial dilutions of known concentrations of template DNA measured with the Qubit dsDNA HS kit (Life Technologies, Carlsbad, CA, United States) before each reaction. For *T. phagedenis, T. pedis*, and *T. medium*, plasmids containing the species-specific regions as described by Beninger, et al. (4) were used as standards, whereas genomic DNAs were used for the non-*Treponema* qPCR assays as described by Caddey, et al. (5). After obtaining the copy numbers per μL of sample in the reaction, the value was multiplied by the elution volume to generate the copy numbers per sampling method. Negative controls were included in each reaction (extraction controls and non-template controls).

**Table 1:** Primer and probe sequences for *Treponema* and non-*Treponema* species-specific multiplex and singleplex qPCRs.

**Species**	**Forward primer**	**Reserve primer**	**Probe**	**Study**
*Treponema phagedenis*	CCCGCAGGAAGGTATAATC	CACAGCTGTTGTGGTATTAAG	HEX^®^/ AATCCGCCTACGACTGCGATACCA/ IB^®^FQ	
*Treponema pedis*	ACACCGATTGTACTGAATGA	CCACGAGCTTTCTACAGATT	6-FAM^®^/ ACTACACGTGGAGTACCGAATGCT/ IB^®^FQ	4
*Treponema medium*	AAAGCGCTACGAATCCTAAG	ATCATTACCCGTCCACAAAG	CAL Fluor^®^ Red 610/ TGCACCCTTGTTTACTACTGCACAGCC/ BHQ-2	4
*Porphyromonas levii*	GGGTGTAGTGCCTACAATAG	CCTGAGAAGAGCAGATAGTG	TxR^®^-X NHS/ CTTGTCACCATCAAAGGCGGCG/IB^®^FQ	5
*Bacteroides pyogenes*	ATTGGCGCTTGTCTCCTACC	TATTCATCCATCGTGCGGCC	6-FAM^®^/ CTGACAGACGAAACCCTCAGCAGAATACT/ IB^®^FQ	5
*Fusobacterium* sp.^*^	TCTTTCAA TGCTGGGA TGCTCT	TGATGGTCCACAATTCTCTCTACA	HEXTM/ CTCACTTTTGCACTTATTTCCTGCACTGA/ IB^®^FQ	5
*Fusobacterium necrophorum*	AACCTCCGGCAGAAGAAAAATT	CGTGAGGCATACGTAGAGAACTGT	6-FAM^®^/ TCGAACATCTCTCGCTTTTTCCCCGA/ BHQ-1	16

* 97% identity with F. mortiferum.

### Statistical analysis

Copy numbers of bacterial DNA in M2 DD lesions collected by biopsy, FNA, and swab were log_10_-transformed and compared for each species using the Kruskal-Wallis test, followed by Dunn's post–hoc test to evaluate which method is significantly different from each other. To evaluate whether the comparison between samples collected from the center of the lesion with swabs was coherent with the biopsy collection on the edge of the lesion, log copy numbers per mg of tissue in each paired biopsy (edge vs. center) were compared using the Wilcoxon rank test. To investigate the relationship between methods, Spearman's correlation coefficient was used. To assess the agreement between the sampling methods using quantitative data, Lin's Concordance Correlation (CCC) coefficient was calculated, and Bland-Altman plots were produced on continuous data (log copy numbers of bacterial DNA). Lin's CCC measures agreement and ranges from−1 to 1, with 1 being a perfect agreement and−1 a perfect disagreement, and 0 no agreement between the predicted and observed values (17). Bland-Altman plots depict the difference found between the methods plotted against the mean, and a good agreement means that 95% of the data points are within ±2 standard deviations (SD) of the mean difference, representing the upper and lower limits of agreement (LoA) (18). For categorical data (bacterial presence or absence), contingency tables were created to test reliability among the sampling methods agreement using Cohen's Kappa coefficient, which ranges from 0 to 1. The following interpretation was used: κ = 0 indicated no agreement, κ = 0.01–0.2 as slight, κ = 0.21–0.40 as fair, κ = 0.41–0.60 as moderate, κ = 0.61–0.80 as substantial, and κ = 0.81–1.00 as almost perfect agreement (19). All the analyses were performed using R v2022.02.3 (RStudio, Boston, MA, United States) and were considered statistically significant when *p* < 0.05.

## Results

**Figure 1:**
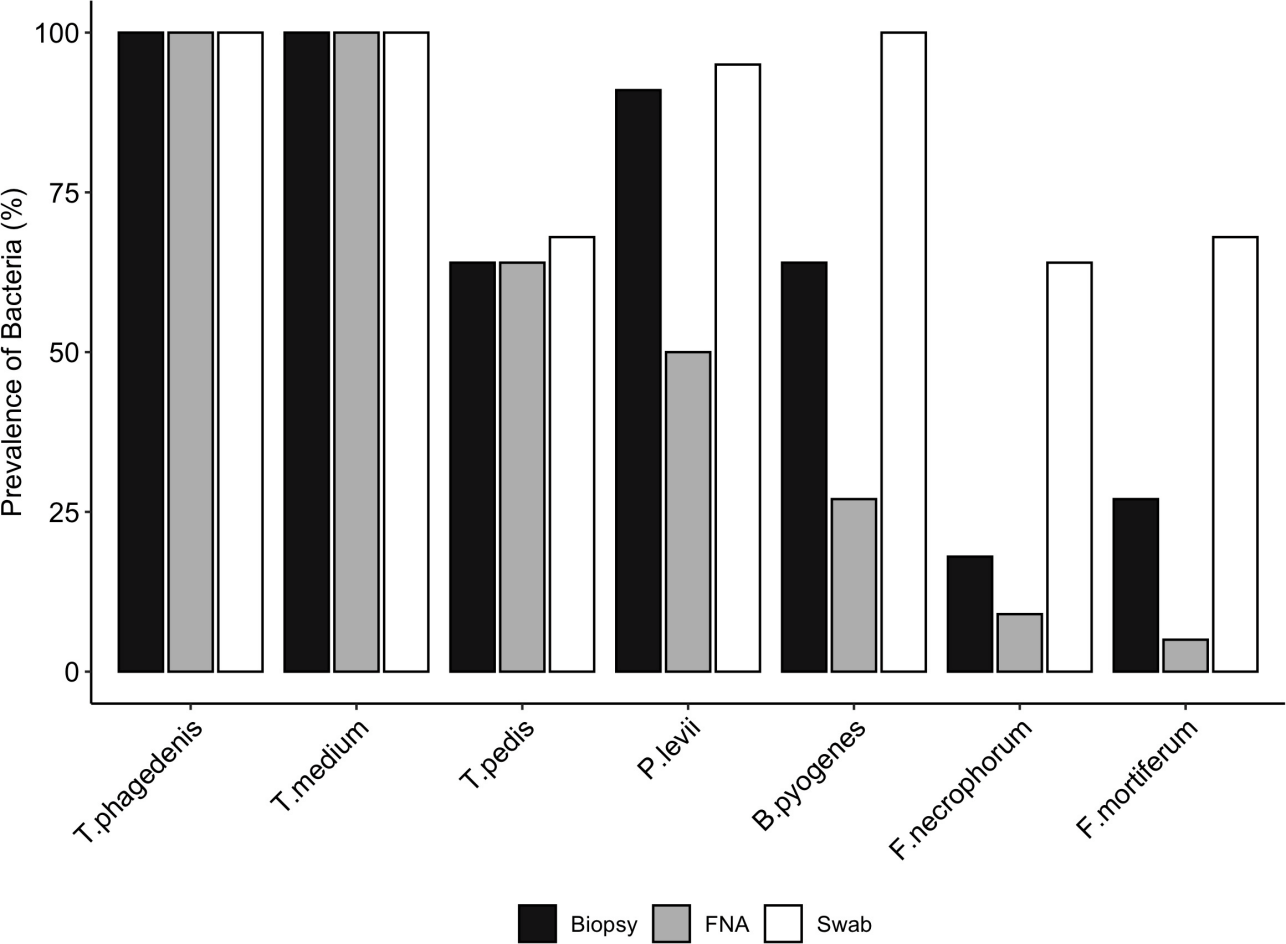
Prevalence (%) of each bacterial species presence in biopsies, swabs, and fine-needle aspiration (FNA) from M2 DD lesions detected by qPCR. A McNemar's test indicated significant differences in the proportions of the presence of *Bacteroides pyogenes, Fusobacterium mortiferum*, and *Fusobacterium necrophorum* between biopsies and swabs, and *Porphyromonas levii, Bacteroides pyogenes*, and *Fusobacterium mortiferum* between biopsies and aspirates (*p* < 0.01); however, no significant difference was observed for the other species.

**Figure 2:**
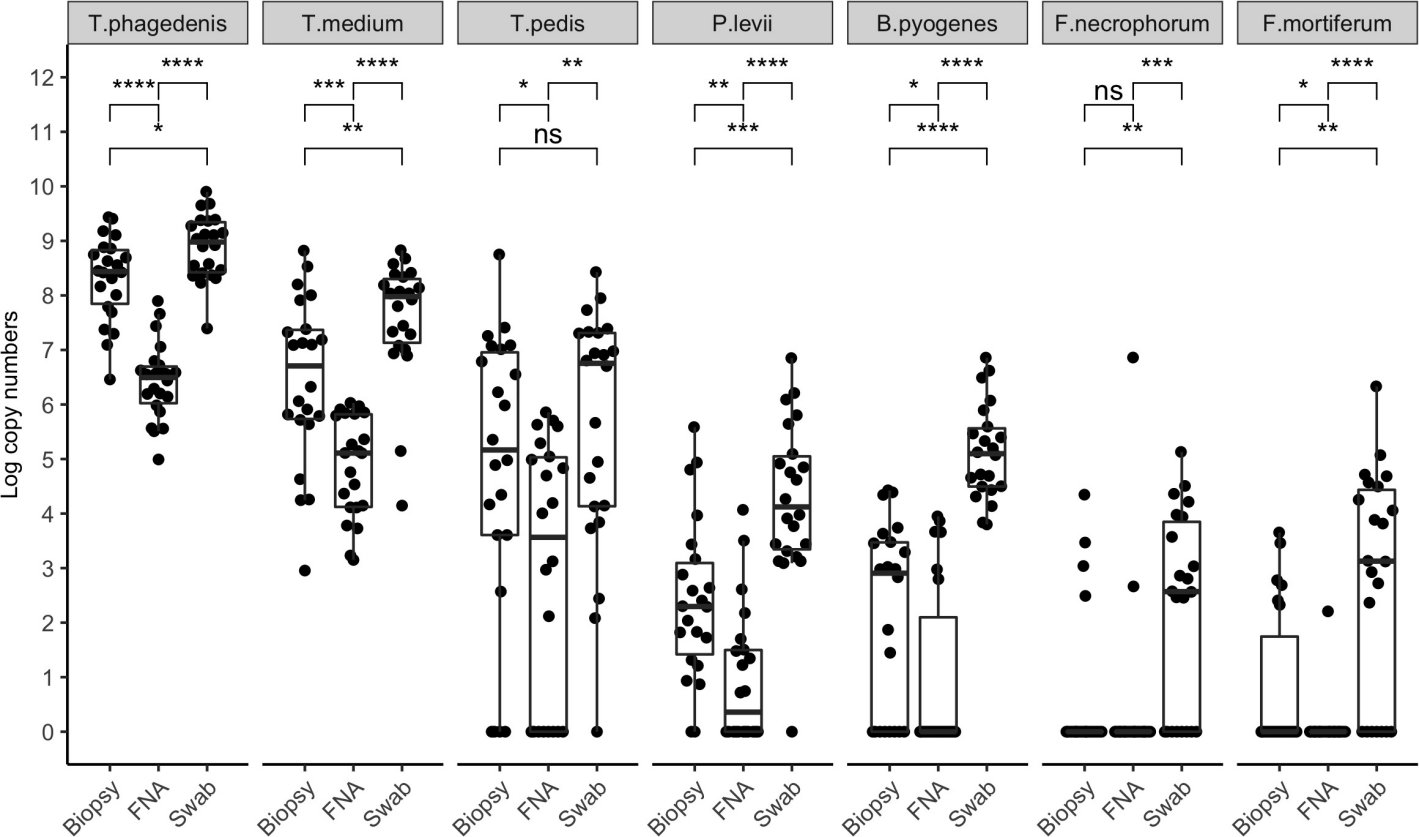
Bacterial quantification (log copy numbers) by species for each sampling method. Boxplot showing the distribution of data, with each dot representing one sample (*n* = 22). Related *p*-values for Dunn's post–hoc test among the sampling methods: biopsy, fine-needle aspiration (FNA), and swab for *Treponema phagedenis, Treponema medium, Treponema pedis, Porphyromonas levii, Bacteroides pyogenes, Fusobacterium necrophorum*, and *F. mortiferum*. No significant difference was observed for *T. pedis* between biopsy and swabs, and *F. necrophorum* in biopsy and aspirates only. Asterisks and horizontal bars indicate differences in bacterial quantification among the sampling methods: ns: *p* > 0.05, *p < 0.05, **p < 0.01, ***p < 0.001, ****p < 0.0001.

Bacterial quantification was compared between two paired biopsies (edge vs. center, *n* = 11), and no significant difference in log copy numbers was observed for all species. The average bacterial DNA log copies and standard deviations recovered from edge biopsies was 8.1±0.7, 6.6 ±2.4, 5.3 ±2.9, 2.5 ±1.7, 2.3 ±1.8, 0.5 ±0.8, and 1.1 ±1.6, *T. phagedenis, T. medium, T. pedis, P. levii, B. pyogenes, F. necrophorum*, and *F. mortiferum*, respectively, whereas from center biopsies was 8.3 ±0.7, 6.9 ±1.8, 5.6 ±3.0, 2.6 ±1.7, 2.2 ±1.9, 0.7±1.1, and 0.4 ±0.9 for *T. phagedenis, T. medium, T. pedis, P. levii, B. pyogenes, F. necrophorum*, and *F. mortiferum*, respectively. The percentage of samples positive for the different target bacteria in DD M2 lesions collected using a punch biopsy, FNA, or swabs based on qPCR is shown in Figure 1. Swabs were more often positive than biopsies and FNA for all target species except for *T. phagedenis* and *T. medium*, in which the prevalence was 100% (22/22) in all sampling methods. Conversely, aspirates were more often negative for all target species. The frequency of detection of *T. pedis, P. levii, B. pyogenes, F. necrophorum*, and *F. mortiferum* was lower in biopsies than swabs, decreasing from 68% (15/22) to 64% (14/22), 95% (21/22) to 91% (20/22), 100% (22/22) to 64% (14/22), 64% (14/22) to 18% (4/22), and 68% (15/22) to 27% (6/22) in swabs and biopsies, respectively. Comparing FNA to biopsies, *T. pedis* was detected in the same proportion, whereas *P. levii* detection in biopsies increased from 50% (11/22) to 91% (20/22), *B. pyogenes* from 27% (6/22) to 64% (14/22), *F. necrophorum* from 9% (2/22) to 18% (4/22), and *F. mortiferum* from 5% (1/22) to 27% (6/22). Swabs of M2 lesions yielded more bacterial cells for all target species than biopsies, while fewer bacterial cells were recovered from aspirates (Figure 2). The average log copy numbers and standard deviations recovered from biopsies was 8.3 ±0.7, 6.5 ±1.5, 4.0 ±3.3, 2.4 ±1.5, 2.1 ±1.7, 0.6 ±1.3, and 0.8 ±1.3 for *T. phagedenis, T. medium, T. pedis, P. levii, B. pyogenes, F. necrophorum*, and *F. mortiferum*, respectively, whereas from swabs was 8.9 ±0.6, 7.6 ±1.1, 4.6 ±3.4, 4.2 ±1.5, 5.1 ±0.9, 2.2 ±1.8, and 2.7 ±2.1 and from FNA was 6.4 ±0.7, 4.9 ±0.9, 2.9 ±2.4, 1.0 ±1.2, 1.0 ±1.6, 0.3 ±1.0, and 0.1 ±0.5 for *T. phagedenis, T. medium, T. pedis, P. levii, B. pyogenes, F. necrophorum*, and *F. mortiferum*, respectively. The number of *T. phagedenis, T. medium, P. levii, B. pyogenes*, and *F. mortiferum* cells detected by qPCR was significantly different among all sampling methods; however, no difference was observed between the number of *T. pedis* recovered from lesion biopsies and swabs. When biopsies were compared to FNA, no difference was observed for *T. pedis* and *F. necrophorum* quantities. *T. phagedenis* was the most abundant species in all sampling techniques, whereas *F. mortiferum* was the least abundant in either biopsy or FNA, and *F. necrophorum* in swabs.

**Figure 3:**
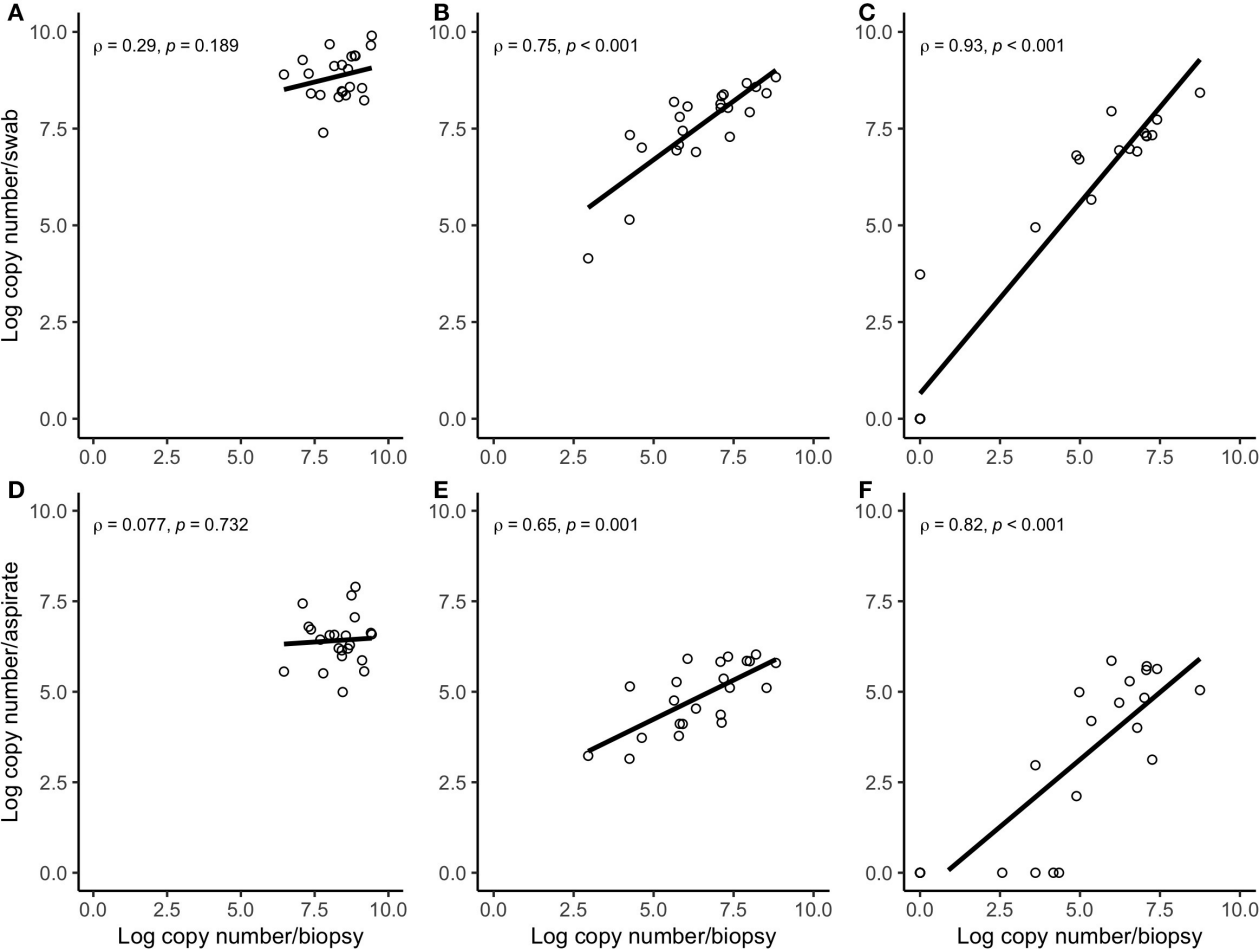
Spearman correlation plots between the amount (log-transformed copy numbers) of *Treponema phagedenis* **(A)**, *Treponema medium* **(B)**, *Treponema pedis* **(C)** in swabs and biopsies of bovine DD active M2 lesions, and *T. phagedenis* **(D)**, *T. medium* **(E)**, and *T. pedis* **(F)** in aspirates and biopsies of bovine DD active M2 lesions. There were significant correlations between the quantity of *T. pedis* and *T. medium* in biopsies and swabs and biopsies and aspirates. There were no significant correlations between the quantity of *T. phagedenis*, neither in biopsies and swabs nor in biopsies and aspirates.

**Figure 4:**
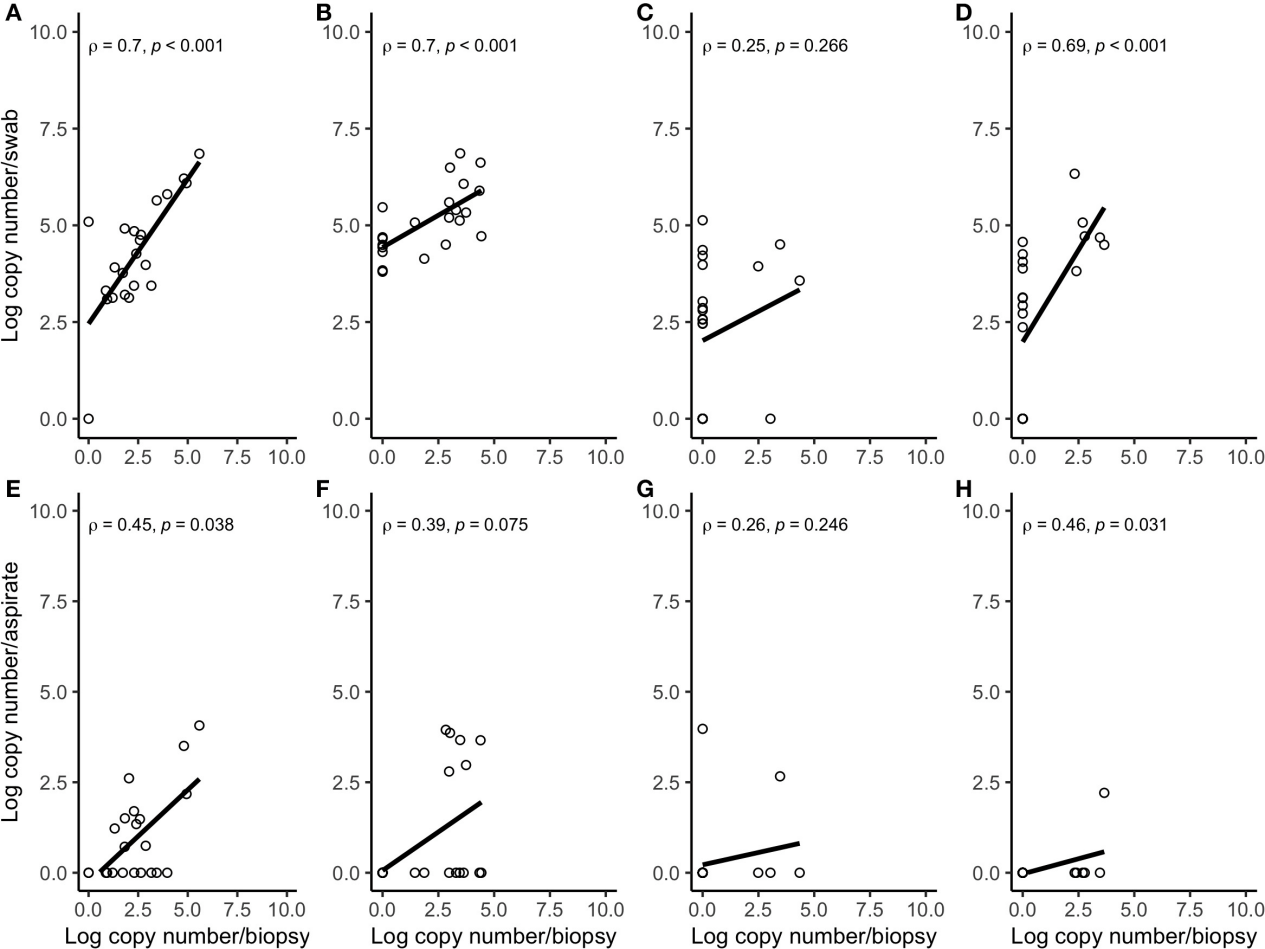
Spearman correlation plots between the amount (log-transformed copy numbers) of *Porphyromonas levii* **(A)**, *Bacteroides pyogenes* **(B)**, *Fusobacterium necrophorum* **(C)**, and *Fusobacterium mortiferum* **(D)** in swabs and biopsies of bovine DD active M2 lesions, and *P. levii* **(E)**, *B. pyogenes* **(F)**, *F. necrophorum* **(G)**, and *F. mortiferum* **(H)** in aspirates and biopsies of bovine DD active M2 lesions. There were significant correlations between the quantity of *P. levii, B. pyogenes*, and *F. mortiferum* in biopsies and swabs and *P. levii, F. mortiferum* in biopsies and aspirates. There were no significant correlations between the quantity of *F. necrophorum*, neither in biopsies and swabs nor in biopsies and aspirates and *B. pyogenes* in biopsies and aspirates.

Correlation coefficient and Lin's CCC among the sampling methods for each species are given in Table 2. Correlations between the number of the target bacterial species in swabs, aspirates, and biopsies were investigated by calculating Spearman's rho (ρ) correlation coefficient. Strong positive correlations were observed between the quantity of *T. pedis* and *T. medium* in biopsies and both less-invasive sampling methods. No significant correlations were observed for *T. phagedenis* when biopsies were compared to swabs and FNA (Figure 3). For the non-*Treponema* species, strong positive correlations between the number of *P. levii* and *F. mortiferum* in biopsies and both less-invasive sampling methods were observed and between the number of *B. pyogenes* in biopsies and swabs. No significant correlations were observed for *F. necrophorum* when biopsies were compared to swabs and FNA and for *B. pyogenes* in biopsies and FNA (Figure 4). Lin's CCC was also calculated to assess agreement between the log copy number of the target species in both sampling methods. A positive agreement was observed between bacterial quantification in biopsies and swabs, having *T. pedis* with the highest coefficient, followed by *T. medium, P. levii, B. pyogenes, F. mortiferum, T. phagedenis*, and *F. necrophorum*. When bacterial numbers were compared between biopsies and FNA, the highest coefficient was observed for *T. pedis*, followed by *T. pedis, P. levii, B. pyogenes, T. medium, F. mortiferum, F. necrophorum*, and *T. phagedenis*.

**Table 2:** Spearman's correlation coefficient, Lin's Concordance Correlation Coefficient, and Bland-Altman results of bacterial quantification of each species in DD M2 lesions (*n* = 22) among the sampling methods.

**Bacterial species**	**Compared sampling method to biopsy**	**rho (ρ)**	**CCC [95% CI]**	**Bland-Altman [mean difference, (upper, lower limits of agreement)]**
*Treponema phagedenis*	Swab	0.29	0.17 [−0.14, 0.46]	−0.54 (−2.22, 1.14)
	FNA	0.08	0.01 [−0.08, 0.11]	1.90 [−0.10, 3.89]
*Treponema medium*	Swab	0.75^**^	0.57 [0.34, 0.74]	−1.12 (−2.84, 0.60)
	FNA	0.65^**^	0.35 [0.14, 0.52]	1.59 [−0.54, 3.71]
*Treponema pedis*	Swab	0.93^**^	0.94 [0.87, 0.97]	−0.59 (−2.49, 1.30)
	FNA	0.82^**^	0.66 [0.42, 0.81]	1.80 [−1.18, 4.78]
*Porphyromonas levii*	Swab	0.70^**^	0.42 [0.19, 0,60]	−1.85 (−3.89, 0.18)
	FNA	0.45^*^	0.38 [0.12, 0.59]	1.44 [−0.94, 3.81]
*Bacteroides pyogenes*	Swab	0.69^**^	0.34 [0.10, 0.54]	−3.04 (−5.66,−0.40)
	FNA	0.39	0.37 [0.04, 0.64]	1.14 [−2.28, 4.55]
*Fusobacterium necrophorum*	Swab	0.25	0.13 [−0.14, 0.40]	−1.60 (−5.57, 2.38)
	FNA	0.26	0.17 [−0.23, 0.52]	0.30 [−2.68, 3.29]
*Fusobacterium mortiferum*	Swab	0.69^**^	0.33 [0.10, 0.53]	−1.95 [−5.20, 1.31]
	FNA	0.46^*^	0.24 [0.03, 0.43]	0.69 [−1.65, 3.02]

FNA, fine-needle aspiration; rho (ρ), Spearman's correlation coefficient; CCC, Linn's Concordance Correlation Coefficient; CI, Confidence interval.

** : Correlation is significant at the 0.01 level (2-tailed).

* : Correlation is significant at the 0.05 level (2-tailed).

**Figure 5:**
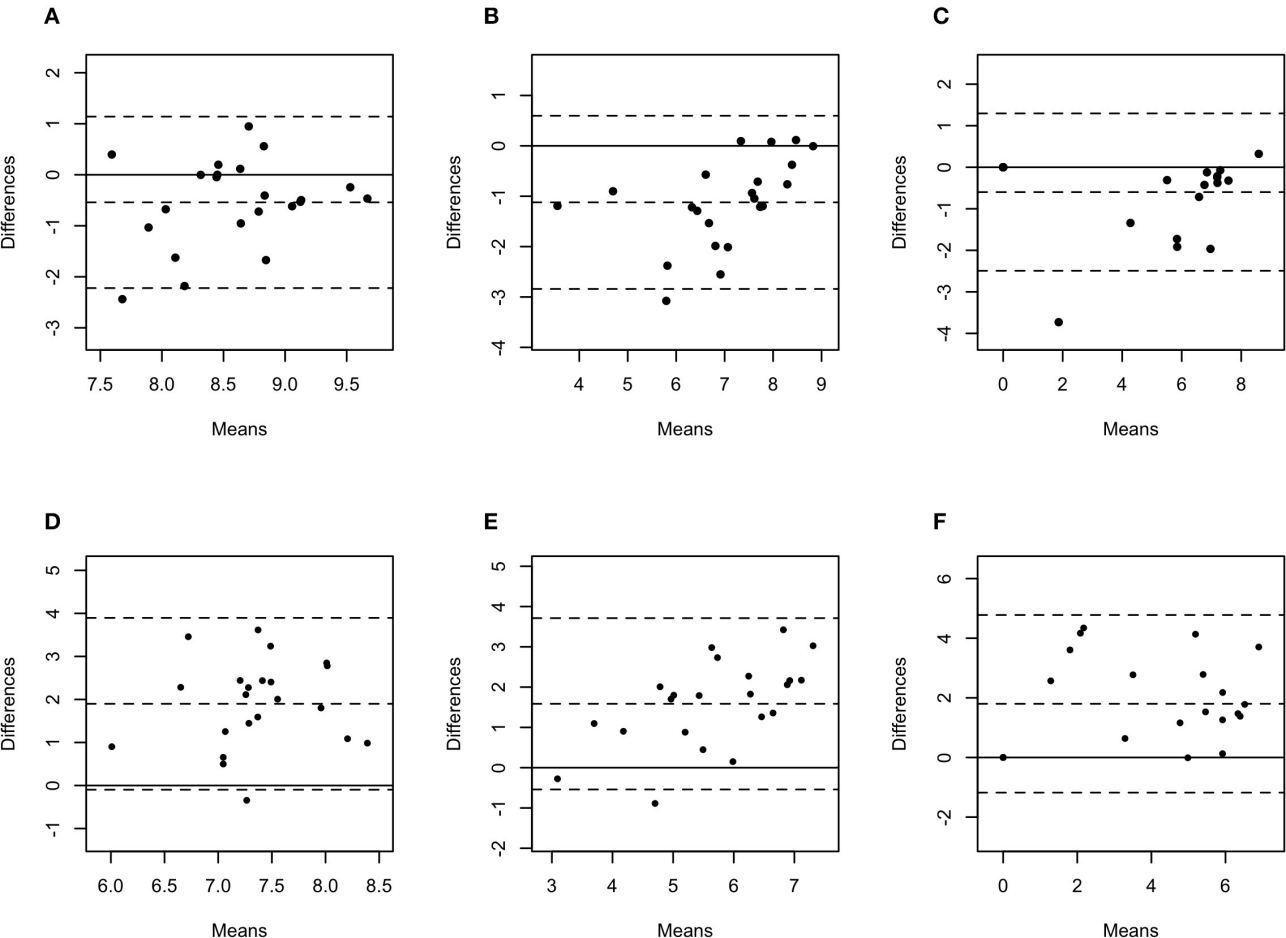
Bland-Altman plots representing the agreement between the quantity of *Treponema phagedenis* **(A)**, *Treponema medium* **(B)**, and *Treponema pedis* **(C)** in biopsies and swabs, and *T. phagedenis* **(D)**, *T. medium* **(E)**, and *T. pedis* **(F)** in biopsies and aspirates. The center dotted represents the mean of difference (bias), and the two contiguous dotted lines represent the upper and lower limit of 95% of agreement. The solid line crossing the axis at zero represents no difference between the sampling methods (perfect agreement; bias = 0).

**Figure 6:**
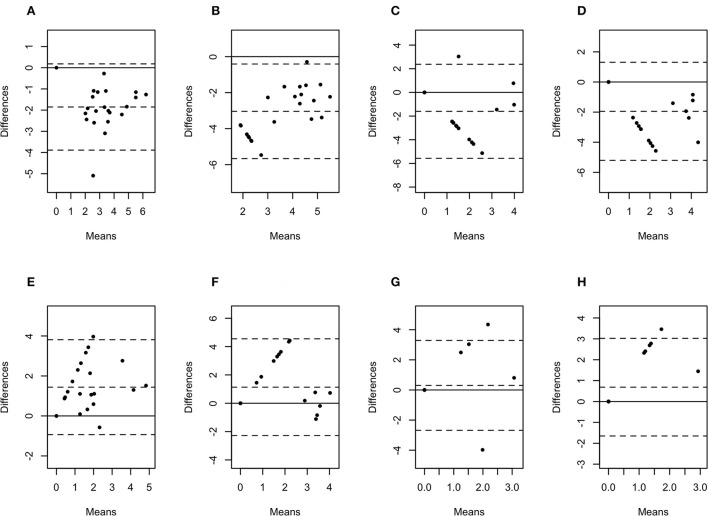
Bland-Altman plots representing the agreement between the quantity of *Porphyromonas levii* **(A)**, *Bacteroides pyogenes* **(B)**, *Fusobacterium necrophorum* **(C)**, and *Fusobacterium mortiferum* **(D)** in biopsies and swabs, and *P. levii* **(E)**, *B. pyogenes* **(F)**, *F. necrophorum* **(G)**, and *F. mortiferum* **(H)** in biopsies and aspirates. The center dotted represents the mean of difference (bias), and the two contiguous dotted lines represent the upper and lower limit of 95% of agreement. The solid line crossing the axis at zero represents no difference between the sampling methods (perfect agreement; bias = 0).

The difference in bacterial enumeration depending on the sampling method was plotted against the mean using a Bland-Altman plot (Figures 5, 6). The difference in log copy numbers comparing biopsies with swabs for each target bacterial species was−0.5 for *T. phagedenis*,−0.6 for *T. pedis*,−1.1 for *T. medium*,−1.6 for *F. necrophorum*,−1.9 for *P. levii*,−2.0 for *F. mortiferum*, and−3.0 for *B. pyogenes*, whereas the comparison between the log copy numbers in biopsies and FNA resulted in the difference of 1.9 for *T. phagedenis*, 1.6 for *T. medium*, 1.8 for *T. pedis*, 1.4 for *P. levii*, 1.1 for *B. pyogenes*, 0.3 for *F. necrophorum*, and 0.7 for *F. mortiferum* (Supplementary material 1). *B. pyogenes* quantification in biopsies and swabs presented the greatest bias from zero, indicating the highest discrepancy between those methods. In contrast, *T. phagedenis* presented the smallest bias from zero. In the biopsies and FNA comparison, *T. phagedenis* presented the greatest bias from zero, and *F. necrophorum* the smallest. Furthermore, based on the LoA width, the widest LoA between biopsies and swabs quantification was observed for *F. necrophorum* followed by *F. mortiferum, B. pyogenes, P. levii, T. pedis, T. medium*, and *T.phagedenis*, in with decreasing order (Table 2). Regarding biopsies and FNA, the widest LoA was detected for *B. pyogenes* followed by *F. necrophorum, T. pedis, P. levii, F. mortiferum, T. medium* and *T. phagedenis*, in with decreasing order.

The qualitative outcomes for the presence/absence of target bacterial species were calculated using Cohen's kappa coefficient of agreement and reliability between biopsies and both less-invasive sampling methods (Table 3). The comparison between biopsies and both less-invasive methods resulted in a perfect agreement for *T. phagedenis* and *T. medium*. For *T. pedis*, the agreement was almost perfect between biopsies and swabs, and substantial between biopsies and FNA. The agreement of the presence in biopsies and swabs was substantial for *P. levii*, fair for *F. mortiferum*, slight for *F. necrophorum*, and null for *B. pyogenes*. In biopsies and FNA, the agreement was fair for *B. pyogenes, F. mortiferum*, and *F. necrophorum*, and slight for *P. levii*. Based on McNemar's test to assess whether the proportions of the presence of the target bacteria in the samples differed between biopsies and the less-invasive methods, a difference was observed for *B. pyogenes, F. necrophorum*, and *F. mortiferum* presence detection in comparison with swabs and observed for *P. levii* and *B. pyogenes* presence detection in comparison with FNA; however, no significant difference was observed for the other bacterial species among the sampling methods.

**Table 3:** Cohen's kappa coefficient and McNemar's *p*-value results of bacterial detection of the presence of each species in DD M2 lesions (*n* = 22) among the sampling methods.

**Bacterial species**	**Compared sampling method to biopsy**	**Agreement (%)**	**Cohen's κ [95% CI]**	**Strength of agreement**	**McNemar's *p*-value**
*Treponema phagedenis*	Swab	100	NA	Perfect	NA
	FNA	100	NA	Perfect	NA
*Treponema medium*	Swab	100	NA	Perfect	NA
	FNA	100	NA	Perfect	NA
*Treponema pedis*	Swab	95.5	0.90 [0.71, 1.09]	Almost perfect	1.0
	FNA	81.8	0.56 [0.17, 0.95]	Substantial	0.125
*Porphyromonas levii*	Swab	95.5	0.64 [−0.03, 1.32]	Substantial	1.0
	FNA	59.1	0.18 [−0.23, 0.59]	Slight	0.0039
*Bacteroides pyogenes*	Swab	63.6	0 [−0.55, 0.55]	Null	0.0078
	FNA	63.6	0.35 [−0.01, 0.71]	Fair	0.0078
*Fusobacterium necrophorum*	Swab	45.5	0.07 [−0.28, 0.43]	Slight	0.0063
	FNA	81.8	0.24 [−0.43, 0.91]	Fair	0.625
*Fusobacterium mortiferum*	Swab	59.1	0.30 [0.06, 0.74]	Fair	0.0077
	FNA	77.3	0.23 [−0.37, 0.82]	Fair	0.0625

FNA, fine-needle aspiration; κ, Cohen's kappa coefficient; CI, Confidence interval; NA, non-applicable.

## Discussion

Using species-specific qPCR, swabs and aspirates of M2 DD lesions efficiently detect the presence of *T. phagedenis, T. pedis*, and *T. medium* at a similar frequency and in good agreement with lesion biopsies, but little agreement was observed for the detection of *P. levii, B. pyogenes, F. necrophorum, F. mortiferum* presence, which were more often present in swabs. Swabs collected higher bacterial loads than biopsies, with the smallest difference in quantity observed for the target *Treponema* species and the largest discrepancy for the non-*Treponema* anaerobes. The quantity of *T. phagedenis, T. medium*, and *T. pedis* was larger in swabs and smaller in aspirates when compared to biopsies; however, the differences in bacterial enumeration observed between biopsies and swabs are smaller than in biopsies and aspirates. Hence, the results from this study suggest that swabs provide a simpler and less-invasive technique for detecting the presence of treponemes in DD M2 lesions using qPCR. Because of the greater discrepancies in the number of bacteria recovered from biopsies and FNA, only swabs were effective in quantifying amounts of treponemes in DD lesions similar to biopsies, and aspirates were less suitable for bacterial quantification compared to other methods. However, taking into account that there is not enough evidence to attribute specific roles to each of the DD-associated bacteria as secondary or primary invaders, the greater disagreement among sampling methods might indicate different niches for *Treponema* and non-*Treponema* species in the lesion. *T. phagedenis* was the most prevalent species, matching the results observed in earlier studies in M2 lesions in dairy (4) and beef cattle (5). However, *T. medium* was more often detected than the previously reported quantities in lesions of the same stage (M2) in beef cattle, while *T. pedis* was detected in similar quantities, suggesting there may be differences in microbial community structure due to distinct housing and management practices between dairy and beef cattle operations.

Previous investigations of treponemes involved in DD using swabs detected them on hoof knifes (20, 21), DD lesions in captive European bison (22), and Contagious Ovine Digital Dermatitis (CODD) lesions experimentally induced in sheep (23); however, those results were based on relative abundance and disregarded total bacterial load, providing information about the presence/absence of bacterial species rather the amount of particular bacterial species. Real-time qPCR enables the amplification of species-specific genes, which are detected by specific fluorescent probes, and the DNA copies enumeration can be calculated using a standard curve produced with serial dilutions of known concentrations of template DNA, converting threshold cycles to the absolute quantity of target gene (24). With this molecular technique, we can increase the knowledge about bacterial quantifies and thereby further investigate their roles in the disease.

Biopsies sampled a more focused area at the lesion edge, capturing the three-dimensional structure of the skin, while swabs sampled only the surface area of lesions, but with a greater exudate absorption capacity. When the microbiome of CODD lesions in sheep was compared between swabs and biopsies, researchers observed a distinct bacterial diversity, with *Spirochetaceae* being more abundant in biopsies and *Porphyromonadaceae* being abundant in both methods (23). Similarly, *Spirochetes* were identified in DD deep biopsies, and *Firmicutes* in superficial strata using 16S rRNA gene sequencing, demonstrating the higher presence of opportunistic pathogens in a superficial skin layer (25). Additional studies investigating the microorganisms involved in udder cleft dermatitis in dairy cows failed to culture treponemes from swab (26), but successfully detected them by nested PCR (27). Since treponemes are difficult to cultivate, a cultured-based method may result in false negative outcomes. Furthermore, previous studies have demonstrated that *Treponema* species are present in different tissue layers in DD lesions based on fluorescent *in situ* hybridization (FISH) (9, 28) and Steiner silver staining (10). *T. phagedenis* has been detected in all skin layers, whereas *T. medium* was detected deeper. Our results have shown the presence of *T. phagedenis* and *T. medium* in 100% of samples using all sampling methods, and *T. pedis* in 68% of the swabs, 64% of aspirates, and 64% of biopsies demonstrating that, although those species are considered invasive, their DNA could be recovered by surface swabs. The M2 DD lesions assessed in this study are ulcerative, hemorrhagic, open wounds that lost completely the stratum corneum (11), which along with the application of sufficient pressure to obtain wound exudate using the swab allowed a greater detection of *Treponema* species DNA in this study. Although lidocaine has been suggested to have antimicrobial activities (29, 30), it can affect microbial activities but not bacterial DNA detection by qPCR (31). Therefore, the use of lidocaine in this study prior to sampling collection is unlikely to affect our results. In addition, active DD lesions can be painful, but not in all cases (32). As a consequence of local anesthesia typically not being required for swabbing DD lesions for research purposes, animals may experience some transient discomfort and pain during the swab sampling. However, the non-invasive nature of this method and the prevention of an open wound after the procedure in the heel bulb of cattle give preference to sampling by swabs rather than biopsies.

Despite the similar frequency of T. *phagedenis, T. pedis*, and *T. medium* in biopsies and FNA, and the usefulness of this technique to detect their presence in DD lesions, their numbers were significantly lower in aspirates. Fluids obtained from FNA are from deeper layers of tissue, being useful for microbiological studies. This method has been used to diagnose soft tissue infections (33–35) and the rate of bacterial DNA recovery differs among these studies depending on the disease characteristics. Because it penetrates deeper into the tissue compared to swab, FNA is considered equivalent to tissue biopsy without being more invasive and may recover more deeply invaded treponemes. However, our results demonstrate that the amount of bacterial DNA recovered from aspirates was significantly lower than that from biopsies and swabs, with a large difference in bacterial enumeration present in the lesion. This may be explained by the small fluid volume recovered from aspirates, and the challenge of recapturing the aspirate from the semi-solid anaerobic transport media prior to DNA extraction.

There was a positive correlation between the amount detected by FNA, swabs, and biopsies for all bacterial species, but this correlation was strong for *T. pedis, T. medium, P. levii, F. mortiferum*, and *B. pyogenes*, and weak for *T. phagedenis* and *F. necrophorum* in swabs and for *T. phagedenis, F. necrophorum*, and *B. pyogenes* in FNA. One important point is that correlation reveals the relationship between the outcomes but does not measure the agreement between sampling methods, meaning that as the log copy numbers of an organism increase in swabs or aspirates, there is an increase in log copy numbers of the same organism in biopsies. As shown in Figures 3, 4, *T. phagedenis, F. mortiferum*, and *F. necrophorum* presented a small variability in quantities, which results in a lower apparent correlation. However, *T. phagedenis* data is distinct from *F. mortiferum* and *F. necrophorum*. Whereas *T. phagedenis* in each sample (100%, 22/22) presented a small range of high log copy numbers in both methods, *F. necrophorum* and *F. mortiferum* presented an equal variability (homoscedasticity) since their log copy numbers in 81% (18/22) and 73% (16/22) of the biopsies, 36% (8/22) and 32% (7/22) of the swabs, and 91% (20/22) and 95% (21/22) of the aspirates was zero. For Lin's CCC, which measures how far the best-fit line deviates from the 45°line (perfect agreement), a small CCC between biopsies and swabs was observed for *T. phagedenis, F. mortiferum*, and *F. necrophorum* and between biopsies and FNA for those three species, suggesting a poorer agreement. Given that CCC also depends on the range of data, these correlation coefficients also need to be interpreted with caution.

Bland-Altman plot analysis was used to measure agreement between biopsies, swabs, and FNA bacterial quantification. Although the correlation observed for *T. phagedenis* was weak, this organism presented the lowest bias between biopsies and swabs through the Bland-Altman plot analysis, suggesting that on average, swabs measured amounts of *T. phagedenis* close to those by biopsies. Conversely, the lowest bias between biopsies and FNA was observed for *F. necrophorum*. A bias equal to zero means that both methods obtained the same bacterial quantity. The observed bias for each target species suggested that on average swabs measured 0.5, 0.6, 1.1, 1.6, 1.9, 2.0, and 3.0 log copies more than biopsies for *T. phagedenis, T. pedis, T. medium, F. necrophorum, P. levii, F. mortiferum*, and *B. pyogenes*, respectively. In contrast, FNA measured on average 1.9, 1.6, 1.8, 1.4, 1.1, 0.3, and 0.7 log copies less than biopsies for *T. phagedenis, T. pedis, T. medium, F. necrophorum, P. levii, F. mortiferum*, and *B. pyogenes*, respectively. Those results emphasize the larger amounts of bacterial DNA recovered from swabs and the smaller numbers recovered from FNA. The LoA representing 95% of the data that were within ±2 SD of the mean difference between biopsies and swabs and biopsies and FNA were wider for non-*Treponema* species than for treponemes, which had the narrowest LoA. Therefore, a better equivalence and agreement can be assumed for treponemes between biopsies and swabs, given their lower bias and LoA. Regarding the comparison between biopsies and FNA, treponemes presented the highest bias but the lowest LoA, emphasizing again the bigger difference in the number of bacterial DNA, but a better agreement than non-*Treponema* species in biopsies and FNA. The main reason for this finding is the high percentage of samples with negative detection results for the non-*Treponema* target species, given by bacterial counts of zero. As a consequence, the difference is zero in the Bland-Altman plot. However, the larger range of 95% of data represents the low agreement between the methods. The question remains if one or two log copy numbers can be considered clinically relevant because the minimum infective dose for DD-associated species and the importance of bacterial doses are unknown. Further research should be undertaken to investigate DD transmission and infective dose to help the interpretation of differences and similarities in sample type agreement.

Bacterial presence or absence analyses between the sampling methods detected agreement and reliability of results for each organism. There was a full agreement for the presence of *T. phagedenis* and *T. medium* in swabs, aspirates, and biopsies since all samples were positive. Although *T. pedis* and *P. levii* had the same proportion of agreement between biopsies and swabs (95.5%), the reliability of the *T. pedis* results was 90%, whereas only 64% for *P. levii*. Therefore, the agreement observed for *P. levii* detection was deemed less than for *T. pedis*. *F. mortiferum*, F. *necrophorum*, and *B. pyogenes* presented fair (30%), slight (7%), or null (0%) reliability results, respectively, despite some degree of agreement. A worse agreement was detected between biopsies and FNA, with reliability being substantial for *T. pedis* (56%), fair for B. *pyogenes* (35%), *F. necrophorum* (24%), *F. mortiferum* (23%), and slight for *P. levii* (18%). *B. pyogenes, F. necrophorum*, and *F. mortiferum* demonstrated the greatest discrepancies between the sampling methods, being more frequently detected in swabs than biopsies. Higher levels of non-*Treponema* organisms in swabs than biopsies and FNA might reflect the high density of those organisms on the lesion surface and their limited involvement in DD as secondary invaders. This argument has been supported by other researchers assessing the spatial distribution in lesions from FISH (7, 36) and the microbiome of DD lesions (6). *P. levii, F. necrophorum* and *B. pyogenes* are considered opportunistic pathogens and normal inhabitants of the rumen, and they have been previously associated with footrot, hepatic abscesses, and uterine infections in cattle (37, 38). However, a study assessing bacterial gene expression in DD lesions reinforces the involvement of *Porphyromonas* and *Bacteroides* in DD initiation, but not *Fusobacterium* (39). Indeed, the lowest detection frequencies and agreements in this study were related to *F. necrophorum* and *F. mortiferum*. Although it is possible that these non-*Treponema* anaerobes were contaminants and found in association with the lesions because of the lack of antiseptic cleaning before sampling, they have been frequently found in DD lesions (5, 6, 28), and they might have a distinct spatial distribution within the lesion, with their niche being more superficial than treponemes. Given our results, the high abundance of non-*Treponema* anaerobes detected from swabs compared to biopsies needs to be taken into consideration. Longitudinal studies into different sampling methods might reveal more differences in the quantification and detection of DD-associated bacterial species.

Some limitations regarding this study should be mentioned. First, we collected swab samples from the center of the lesion without covering a precise surface area; therefore, the comparison was made based on the total DNA yield from the swab cotton head, and the results could not be normalized and expressed in log copy numbers per cm^2^. Further investigation might be required to standardize the sampling collection in a known surface area. Second, the DNA detected by qPCR was not differentiated between viable and dead cells. Next studies might test the use of propidium monoazide (PMA) to treat the samples prior to DNA extraction for live/dead distinction by qPCR. Finally, no healthy tissue samples were obtained in the present study; we targeted only ulcerative, active lesions for comparison. This approach could potentially reveal bias in the results since bacteria from healthy skin or environment could also be detected by qPCR, especially by using surface swabs. Future surveys of healthy skin (stage M0) and other DD lesion stages, such as chronic (stage M4) and early stage (M1), will be necessary to expand the swab/FNA/biopsy comparison and agreement among different stages of the disease. Especially the collection of M1 lesions could be targeted to have a greater range of *T. phagedenis*, which had a lower apparent correlation due to limited variation between the samples.

In conclusion, it seems reasonable to employ swabs and FNA and real-time qPCR to screen for the presence of treponemes in M2 DD lesions. *T. phagedenis, T. medium*, and *T. pedis* were detected similarly in swabs, aspirates, and biopsies, and *P. levii, F. mortiferum, F. necrophorum*, and *B. pyogenes* were detected more frequently in swabs compared to aspirates and biopsies. More bacterial DNA was recovered from swabs, followed by biopsies and aspirates. A good and reliable agreement for bacterial quantification among the methods was observed only when *T. phagedenis, T. medium*, and *T. pedis* were targeted. Their quantities were larger in swabs and smaller in aspirates in comparison to biopsies; however, the differences in bacterial enumeration observed between biopsies and swabs were smaller than between biopsies and aspirates. The less-invasive sampling methods offer the opportunity to sample multiple places in the same animal or monitor DD initiation, treatment effectiveness, and progression in a long-term repeat-sampling study targeting treponemes involved in this disease.

## Data availability statement

The original contributions presented in the study are included in the article/Supplementary material, further inquiries can be directed to the corresponding author.

## Ethics statement

The animal study was reviewed and approved by the University of Calgary Veterinary Services Animal Care Committee (VSACC) under animal care protocol #AC21-0146. Written informed consent was obtained from the owners for the participation of their animals in this study.

## Author contributions

APD and JDB contributed to the conception and design of the study. APD collected and processed the dairy cattle lesion samples, performed all statistical analyses, and wrote the manuscript. JDB edited and reviewed the manuscript. All authors contributed to the article and approved the submitted version.

## Funding

This work was supported by the Natural Sciences and Engineering Research Council of Canada (NSERC) Collaborative Research and Development grants (CRD) to JDB (CRDPJ/536202-2018).

## Conflict of interest

The authors declare that the research was conducted in the absence of any commercial or financial relationships that could be construed as a potential conflict of interest.

## Publisher's note

All claims expressed in this article are solely those of the authors and do not necessarily represent those of their affiliated organizations, or those of the publisher, the editors and the reviewers. Any product that may be evaluated in this article, or claim that may be made by its manufacturer, is not guaranteed or endorsed by the publisher.
